# Isolation of xylose isomerases by sequence- and function-based screening from a soil metagenomic library

**DOI:** 10.1186/1754-6834-4-9

**Published:** 2011-05-05

**Authors:** Nádia Skorupa Parachin, Marie F Gorwa-Grauslund

**Affiliations:** 1 Department of Applied Microbiology, Center for Chemistry and Chemical Engineering, Lund University, P.O. Box 124, SE-221 00 Lund, Sweden; 2 Present affiliation - Laboratório de Biologia Molecular, Universidade de Brasília, 70910-900 Brasília (DF), Brazil

## Abstract

**Background:**

Xylose isomerase (XI) catalyses the isomerisation of xylose to xylulose in bacteria and some fungi. Currently, only a limited number of XI genes have been functionally expressed in *Saccharomyces cerevisiae*, the microorganism of choice for lignocellulosic ethanol production. The objective of the present study was to search for novel XI genes in the vastly diverse microbial habitat present in soil. As the exploitation of microbial diversity is impaired by the ability to cultivate soil microorganisms under standard laboratory conditions, a metagenomic approach, consisting of total DNA extraction from a given environment followed by cloning of DNA into suitable vectors, was undertaken.

**Results:**

A soil metagenomic library was constructed and two screening methods based on protein sequence similarity and enzyme activity were investigated to isolate novel XI encoding genes. These two screening approaches identified the *xym1 *and *xym2 *genes, respectively. Sequence and phylogenetic analyses revealed that the genes shared 67% similarity and belonged to different bacterial groups. When *xym1 *and *xym2 *were overexpressed in a *xylA*-deficient *Escherichia coli *strain, similar growth rates to those in which the *Piromyces *XI gene was expressed were obtained. However, expression in *S. cerevisiae *resulted in only one-fourth the growth rate of that obtained for the strain expressing the *Piromyces *XI gene.

**Conclusions:**

For the first time, the screening of a soil metagenomic library in *E. coli *resulted in the successful isolation of two active XIs. However, the discrepancy between XI enzyme performance in *E. coli *and *S. cerevisiae *suggests that future screening for XI activity from soil should be pursued directly using yeast as a host.

## Background

The soil habitat is an immensely diverse environment. One gram of soil may harbour up to ten billion bacteria belonging to more than 4,000 to 7,000 different species [1], although this value varies according to the soil type [2]. However, only 1% of the soil bacterial flora can be cultivated under standard laboratory conditions [3]. Metagenomics, which consists of the extraction, cloning and analysis of the entire genetic material in a given habitat [4,5], has emerged as a tool for assessing the genetic information from uncultivable microorganisms. Metagenomic libraries are a powerful source for discovering new biological activities and have already been successfully used to isolate novel hydrolytic enzymes such as amylases, cellulases and xylanases [6,7].

The screening of metagenomic libraries, which is a critical step for the successful isolation of new and improved biological activities, can be performed by sequence homology or activity-based assays [8]. In sequence-based screening, the isolation of novel proteins is based on homology searches. Although it impedes the discovery of entirely new gene sequences, it is an efficient method of isolating enzymes with different levels of similarity from previously identified genes. For instance, sequence-based screening from the metagenome of a hindgut microbiota of a wood-feeding higher termite identified 700 domains with homology to the glycoside-hydrolase catalytic site [9]. In activity-based assays, metagenomic libraries are constructed in expression vectors to allow for the isolation of biological activities from entirely new gene sequences that are also successfully expressed in the chosen host.

Activity-based screening methods have been categorized into chromogenic, product detection and growth test assays [10]. Chromogenic assays are commonly used for the detection of hydrolases from metagenomic libraries [6,7], whereas a product detection method has been utilized for the identification of enzymes from a soil metagenome producing porphyrin intermediates [11]. Although it is a high-throughput method, growth-based screening has not been frequently described, as it may be difficult to develop and implement a selection protocol that clearly discriminates growth from nongrowth.

Metagenomic libraries have not yet been utilized for the isolation of novel genes involved in xylose catabolism. Xylose is the second most abundant sugar in nature, and the production of cost-efficient lignocellulosic bioethanol requires its complete fermentation [12]. One xylose catabolic pathway is found in naturally xylose-utilizing bacteria [13] and some fungi [14,15] and includes the isomerisation of xylose to xylulose via xylose isomerase (XI). High ethanol yields from xylose have been reported for *Saccharomyces cerevisiae *strains harbouring actively expressed XI genes [15-18].

In this study, the possibility of isolating xylose catabolic enzymes from a soil metagenomic library was evaluated. Both sequence-based and growth-based screening were utilized. Each method resulted in the isolation of a novel gene sequence coding for a XI. Restoration of growth on xylose was verified in both *Escherichia coli *and *S. cerevisiae *strains by overexpression of the isolated genes in strains lacking the initial xylose catabolic pathway. The advantages and disadvantages of the two screening methods are discussed.

## Results

### Isolation of XI genes from soil metagenome

A soil metagenomic library with an estimated size of 1.26 × 10^5 ^clones was constructed in plasmid pRSETB (Table 1) using DNA extracted from garden compost and screened for XI activity using either sequence analysis or growth assays.

**Table 1:** Plasmids and strains used in this study

Plasmids/strains	Relevant characteristics	Source
Plasmids		
pGEM-T Easy	PCR product cloning, ampR	Promega (Madison, WI, USA)
pRSETB	ampR, T7p-T7t	Invitrogen (Carlsbad, CA, USA)
p426TEF	*URA3 *TEFp-CYC1t	[43]
pRESTB-XIPiromyces	AmpR, T7p-xiPiromyces-T7t	This study
pRSETB-Xym1	AmpR, T7p-*xym1*-T7t	This study
pRSETB-Xym2	AmpR, T7p-*xym2*-T7t	This study
p426TEF-XiPiromyces	*URA3*, p426TEFp-XiPiromyces-CYC1t	This study
p426TEF-Xym1	*URA3*, p426TEFp-*xym1*-CYC1t	This study
p426TEF-Xym2	*URA3*, p426TEFp-*xym2*-CYC1t	This study
		
*Escherichia coli *strains		
ElectroTen-Blue	Kan^R^, Tet^R^	Stratagene (La Jolla, California, USA)
HB101	*ara-14*, *proA2*, *xyl-5*, *leuB6*, *thi-1*	Takara Bio (Otsu, Shiga, Japan)
DH5α	Sm^R^	Life Technologies (Rockville, MD, USA)
TMB2010	HB101 + pRSETB	This study
TMB2011	HB101 + pRSETB-xiPiromyces	This study
TMB2012	HB101 + pRSETB-Xym1	This study
TMB2013	HB101 + pRSETB-Xym2	This study
		
*Saccharomyces cerevisiae *strains		
TMB3044	CEN.PK 2-1C *Δgre3, his3*::*PGK1*p-*XKS1*-*PGK1*t,*TAL1*::*PGK1*p-*TAL1*-*PGK1*t,*TKL1*::*PGK1*p-*TKL1*-*PGK1*t, *RKI1*::*PGK1*p-*RKI1*-*PGK1*t, *RPE1*::*PGK1*p-*RPE1*-*PGK1*t,*ura3*	[26]
TMB3363	TMB3044, p426TEF	This study
TMB3359	TMB3044, p426TEF-XiPiromyces	This study
TMB3364	TMB3044, p426TEF-Xym1	This study
TMB3365	TMB3044, p426TEF-Xym2	This study

**Figure 1:**
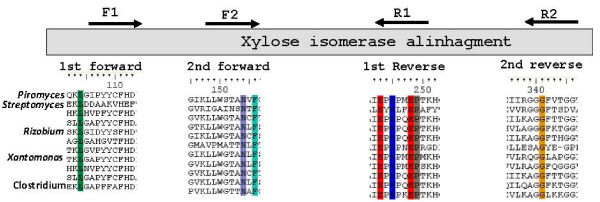
**Conserved regions utilized for the construction of the degenerate primers**. The choice of regions was based on multiple alignments of XI amino acid sequences. The microorganisms chosen, in presented order, are *Piromyces sp*., *Streptomyces diastaticus*, *Escherichia coli*, *Pseudomonas sp*., *Rhizobium sp*., *Streptomyces coelicolor*, *Saccharophagus sp*., *Enterobacter sp*., *Mesorhizobium loti *and *Clostridium sp*.

For the sequence-based screening, degenerate primers were designed based on the alignment of amino acid sequences of known XIs from soil microorganisms such as *Streptomyces sp*. and *Rhizobium sp. *Amino acid sequences of XI genes whose expression has been attempted in *S. cerevisiae*, such as the ones from *Piromyces sp. *[14] and *E. coli *[19], were also included (Figure 1). The first polymerase chain reaction (PCR) was performed with primers DF1 and DR2 (Table 2) and with the soil metagenomic library used as a template. A fragment of approximately 750 bp was amplified. The purified fragment was then utilized as the template for a nested PCR with internal primers DF2 and DR1. A single fragment of about 350 bp was obtained, purified from an agarose gel and cloned into the pGEM-T Easy Vector System (Promega, Madison, WI, USA) (Table 1). Sequence analysis revealed that this fragment belonged to a gene coding for a XI. The complete gene sequence was then obtained by PCR utilizing the metagenomic library as the template and primers designed to the sequence of the known 350-bp fragment and in plasmid pRSETB. The gene sequence encoded a 443-amino acid protein with 83% identity with *Sorangium cellulosum *XI and was designated *xym1*.

**Table 2:** Primers used^a^

Primer	Sequence
DF1	5' AAGCT**(A/C/G/T)**GG**(A/C/G/T)**G**(C/T)(A/C/G/T)**CCT T**(C/T)(C/T)**TATTGTTT**(C/T)**CACGAC 3'
DR2	5' TCCTCC**(A/C/G/T)**TGCTG**(C/T)(T/A)(T/A)**GCCGCC**(A/C/G/T)**CC**(A/C/G/T)**CG**(A/C/G/T)**AG**(A/G)**AT 3'
DF2	5' GGT**(A/G)**T**(A/C/G/T)**AA**(A/G)**CT**(A/C/G/T)**CTCTGGGG**(A/C/G/T)**CAGGCCAA**(C/T)**CT**(A/C/G/T)**TT**(C/T) **3'
DR1	5' **(A/G)**TG**(C/T)**TTCTGGGG**(C/T)**TC**(C/T)**TGCGGCTTGGGCTC **(A/C/G/T)**A**(T/G) **3'
pRSETBF	5' GGTGGACAGCAAATGGGTCGG 3'
pRSETBR	5' GGGCTTTGTTAGCAGCCGGATC 3'
XYM1F	5' CCTGGATCCATGAGCGTTGTTCTTGGCGACAAAG 3'
XYM1R	5' TGGGTCGACTTACCGGATCCACCGGTTCATAATG 3'
XYM2F	5'CAGGAATTCATGAAACTTACCGTAGGAGACAAGG 3'
XYM2R	5' TGGGTCGACTTAAATAAACCTGCTAATCAAATTCTCAATATAC 3'

^a^Restriction sites, when present, are underlined.

**Figure 2:**
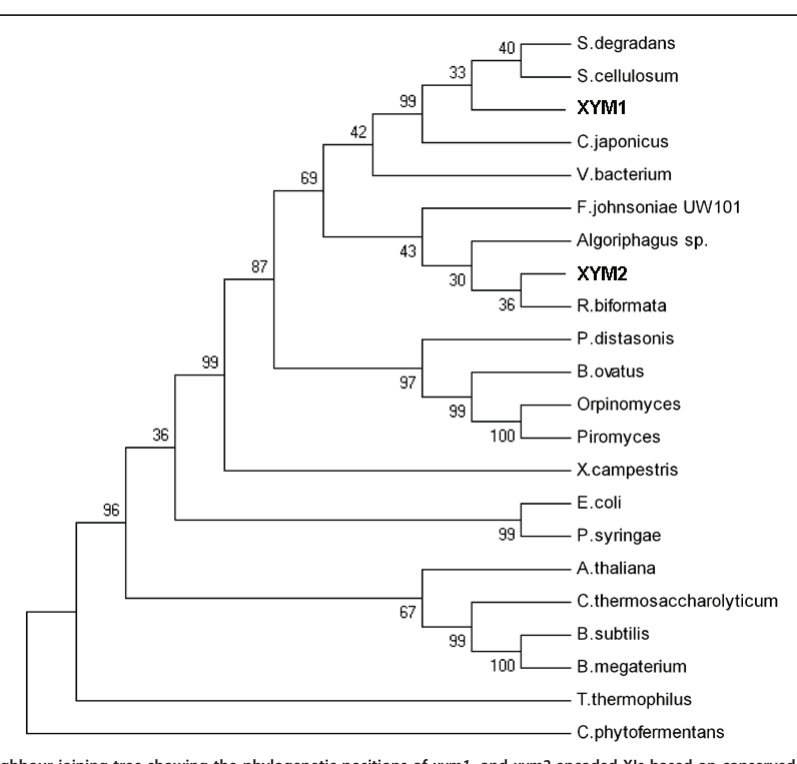
**Neighbour-joining tree showing the phylogenetic positions of *xym1*- and *xym2*-encoded XIs based on conserved amino acids of different XIs**. Microorganism sources of XIs were chosen based on sequence similarity and/or active expression in *Saccharomyces cerevisiae*. The phylogenetic tree was constructed with MEGA software using an unweighted group method with arithmetic mean (UPGMA) bootstrapped with 6,000 interactions.

For the activity screening, the metagenomic library was transferred to the host strain HB101 (Table 1) that has a nonfunctional *xylA*, which allows selection for growth on xylose. After bacterial transformation, cells were plated in defined media supplemented with xylose and incubated at 37°C for four to ten days. Positive (pRSETB-Xi *Piromyces*) and negative (pRSETB) controls were also transformed into the screening strain and plated in selective media to validate the screening method. Plasmids were extracted and sequenced from colonies that grew on the selective media. All extracted plasmids contained identical inserted fragments with an insert size of approximately 2.5 kb. Sequencing analysis revealed a complete open reading frame that corresponded to a XI gene named *xym2*. *xym2 *encoded a protein of 442 amino acids with 73% similarity to the XI from *Robiginitalea biformata *and 67% identity with *xym1*-encoded XI. A phylogenetic tree constructed using MEGA4 software [20] and 22 different XI amino acid sequences revealed that *xym1*-encoded XI was most similar to XIs from the Proteobacteria, while *xym2-*encoded XI was more similar to the XIs from members of the *Bacterioides *phylum (Figure 2).

### Growth evaluation in *E. coli*

The *xym1 *and *xym2 *genes and the positive control *xylA *gene from *Piromyces *[14] were each cloned into the multicopy vector pRSETB, generating plasmids pRSETB-Xym1, pRSETB-Xym2 and pRSETB-XiPiromyces (Table 1). Strain HB101 was transformed with each of the three constructs, as well as the negative control pRSETB, to generate the four strains: TMB2010 (negative control), TMB2011 (*Piromyces xylA*), TMB2012 (*xym1*) and TMB2013 (*xym2*).

**Figure 3:**
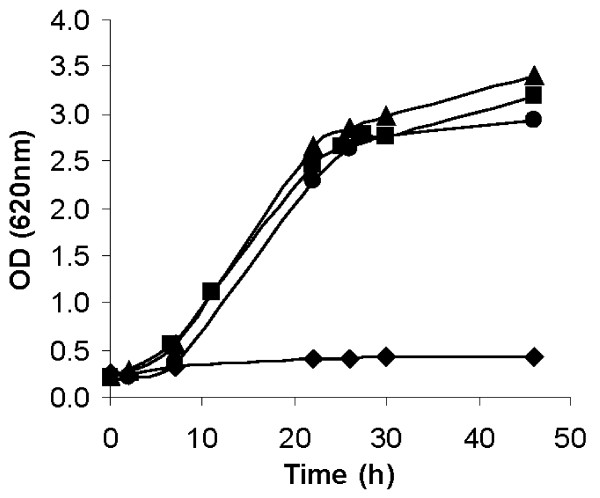
**Aerobic growth of *E. coli *recombinant strains carrying pRSETB-Xym1 (filled triangle), pRSETB-Xym2 (filled circle), pRSETB-XIPiromyces (filled square), or the empty plasmid pRSETB (filled diamond)**. All strains were cultivated at 37°C in SM3 media supplemented with 30 g/L xylose.

Growth was evaluated for the four strains in SM3 liquid media with xylose. Growth with xylose as the sole carbon source was reestablished for all strains overexpressing any of the XI-encoding genes (Figure 3). No significant differences in growth rates and final Optical density (OD) were observed between the positive control strain, TMB2011, and the strains overexpressing *xym1 *and *xym2 *that were isolated from the metagenomic library (Figure 3).

### Growth evaluation in *S. cerevisiae*

**Figure 4:**
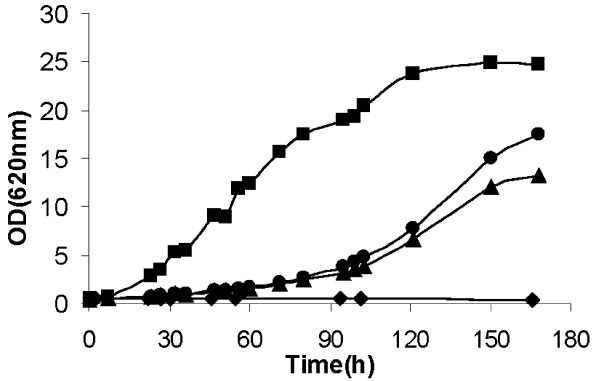
**Aerobic growth of *S. cerevisiae *recombinant strains carrying p426TEF-Xym1 (filled triangle), p426TEF-Xym2 (filled circle), p426TEF-XiPiromyces (filled square) or the empty plasmid p426TEF (filled diamond)**. All strains were cultivated at 30°C in mineral media supplemented with 50 g/L xylose.

XI is a key enzyme for the construction of recombinant S. *cerevisiae *strains for xylose conversion to ethanol. However, despite many efforts [19,21-24], only a few XI genes have been successfully expressed in *S. cerevisiae *at sufficient levels to allow growth on xylose [15,16,25]. Therefore, *xym1 *and *xym2 *were overexpressed in *S. cerevisiae *to assess growth in a recombinant *S. cerevisiae *strain lacking the initial xylose catabolic pathway. *xym1*, *xym2 *and the *xylA *gene from *Piromyces sp. *(positive control) were cloned in the multicopy vector p426TEF to generate plasmids p246TEFXiPiromyces, p426TEF-Xym1 and p426TEF-Xym2 (Table 1). The *S. cerevisiae *screening strain TMB3044 that has been optimised for efficient pentose utilization, but which lacks the initial conversion step from xylose to xylulose [26], was transformed with the three constructed plasmids in addition to the empty plasmid to generate strains TMB3363(empty plasmid), TMB3359(XiPiromyces), TMB3364 (*xym1*) and TMB3365 (*xym2*). No growth was observed for the negative control. In contrast, overexpression of both *xym1 *and *xym2 *enabled growth on xylose in *S. cerevisiae *(Figure 4). However, the strains carrying either *xym1*or *xym2 *grew at a lower growth rate (0.021 hour^-1 ^for both strains) than the positive control harbouring *Piromyces sp *XI (0.069 hour^-1 ^± 0.006) (Figure 4).

### Evaluation of XI-specific activities in both hosts

XI-specific activities were compared in cell extracts of *E. coli *and *S. cerevisiae *cells grown in defined medium with glucose. For each microorganism, positive (*Piromyces sp. *XI) and negative (empty vector) control strains were included for comparison with the strains overexpressing either *xym1 *or *xym2*.

**Figure 5:**
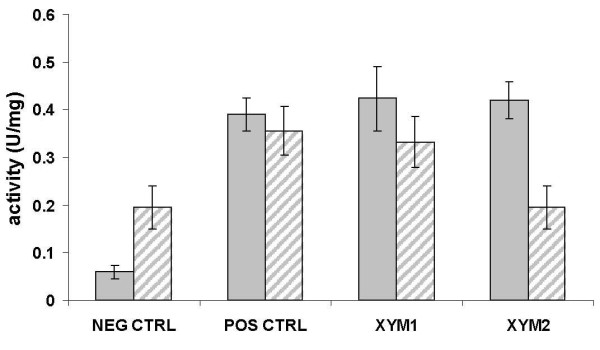
**XI specific activity (U/mg protein^-1^) in *E. coli *(gray) and *S. cerevisiae *(stripped) strains overexpressing *xym1*, *xym2 *and *Piromyces sp*. XI gene**. The background activity was obtained from the same strains carrying the respective empty vectors.

In *S. cerevisiae*, the background activity measured in the negative control (0.195 U/mg protein^-1^) was twice as high as that measured in *E. coli *(0.059 U/mg protein^-1^) (Figure 5). Strains overexpressing the positive control or *xym1 *had about the same specific XI activity in both *E. coli *and *S. cerevisiae*. For the *xym2*-overexpressing strain, the specific activity (0.420 U/mg protein^-1^) was in the same range as the ranges for the *xym1*-overexpressing strain and the positive control in *E. coli*. In contrast, the specific activity in the *xym2*-overexpressing strain was at the level of the background activity in *S. cerevisiae *(0.195 U/mg protein^-1^) (Figure 5).

## Discussion

In this study, for the first time, two screening methods were established in parallel for the isolation of novel XI-encoding genes from a soil metagenomic library. The sequence-based method using degenerate primers deduced from the alignment of amino acid sequences of several XIs from soil microorganisms rapidly resulted in the isolation of the first XI-encoding gene *xym1 *from a metagenomic library. Growth-based screening was also used for the first time to isolate the XI-encoding gene *xym2 *from soil metagenomic library using an *E. coli *strain lacking XI activity. The two different methods resulted in the isolation of two XI-encoding genes that shared 67% identity at the protein level and could both complement *E. coli *and *S. cerevisiae *strains lacking a xylose catabolic enzyme.

Some examples where the sequence-based screening method has already been used include (1) the identification of genes responsible for the hydrolytic dehalogenation of 4-chlorobenxoate from a metagenomic library constructed from a denitrifying, degrading consortium [27] and (2) the isolation of a polyketide synthase gene from a metagenome constructed from marine sediment in the East China Sea [28]. It is a high-throughput screening method, but one disadvantage is that it selects for gene sequences with close homology to those utilized for the design of the degenerate primers. This was notably exemplified by the isolation of closely related chitinase genes from cultured and uncultured marine bacteria [29].

In contrast, activity-based screening permits the identification of previously unknown gene sequences, as the screening is based on the gene product only. One disadvantage, however, is that activity-based screening that relies on chromogenic or fluorogenic assays often require high-throughput equipment, such as colony pickers, that transfer colonies from plates to the assay system. When possible, it is therefore advisable to develop a growth-based screening method because the method is simple (each library can be plated on a limited number of plates, as very few clones will grow) and enables high-throughput (only the clones displaying the desired activity are able to grow). This method is limited in that a host strain or an experimental setup must be designed that does not enable the host to grow unless the desired activity is present.

Screening by growth has previously been used to isolate genes from a soil metagenomic library conferring Na^+^(Li^+^)/H^+ ^antiporter activity to an antiporter-deficient *E. coli *host strain [30]. Novel DNA polymerase activities have also been isolated from glacial ice metagenomic libraries using a cold-sensitive *E. coli *strain that harboured a mutation in the DNA polymerase domain generating lethality at temperatures below 20°C [31]. Finally, a novel D-3-hydroxybutyrate short-chain dehydrogenase with less than 35% similarity to known proteins with similar activity has been isolated from soil metagenomic libraries using a mutant of *Sinorhizobium meliloti *that was unable to synthesize D-3-hydroxybutyrate [32]. In the present study, *xym1*- and *xym2*-encoded XIs displayed strong similarity in amino acid sequence, although they were isolated by using different methodologies. This may be explained by the fact that most of the XI sequences that were used to identify homologous regions for the sequence-based screening originated from organisms that were know to be present in soil.

Screening for XI-encoding genes by growth versus nongrowth was successfully implemented in *E. coli*, since the expression of *xym1 *and *xym2 *genes could restore growth in xylose minimal media with the same growth rate observed for the positive control overexpressing the *Piromyces *XI gene. However, growth was restored when the same genes were transferred to the heterologous host *S. cerevisiae*, where the activity is required for xylose fermentation to ethanol, the growth rate was four times lower than that observed for *Piromyces *XI. Expression of XI-encoding genes in *S. cerevisiae *is a complex issue [33]. The first one to be expressed at high levels in *S. cerevisiae *was *Piromyces *XI [18], and it occurred after many unsuccessful attempts and limited success with several other bacterial and plant XIs [19,21-24]. Successful expression of XI in *S. cerevisiae *and its growth rate on xylose are not always correlated to sequence similarity or enzymatic activity (Table 3). In our case, *xym1*-encoded XI had the same XI activity as *Piromyces sp *XI, but the growth rate was fourfold lower. In contrast, the specific activity of the *xym2*-encoded XI was in the range of the background activity, but the growth rate was identical to the strain expressing *xym1*. The growth rate was also in the same range when the *Xanthomonas campestris *XI gene was expressed, although the reported activity range was about 20 times lower than that for the *xym1*-overexpressing strain (Table 3). Finally, a high activity level and growth rate were achieved when overexpressing a codon-optimised XI gene from *Clostridium phytofermentans *that shared only 54% similarity with *Piromyces sp. *at the protein level (Table 3) [16]. Differences in kinetic properties may also explain the discrepancies among all these results. For instance, the affinity for xylose may be much higher in *xym2*- than in *xym1*-encoded XI, so that the lower rate of conversion would be compensated for by a higher affinity for the substrate. Alternatively, the novel XI may be more inhibited by xylitol than the one from *Piromyces sp. *or *C. phytofermentans*. We were not able to measure *K*_m _or *K*_i _for the new XIs, because the activities were below the detection limit. Transport may also explain differences in behaviour between *S. cerevisiae *and *E. coli*, since active xylose transporters are present in *E. coli *[34,35] but missing in *S. cerevisiae*.

**Table 3:** Reported specific activity and maximum specific growth rate for XI genes cloned in *Saccharomyces cerevisiae*^a^

Source of XI	Activity (U/mg protein)	Aerobic growth rate on xylose (hour^-1^)	Source	Identity with *xym1 *XI	Identity with *xym2 *XI	Identity with *Piromyces *XI
Metagenome *xym1*^b^	0.33 ± 0.05	0.02	This study	100%	67%	61%
Metagenome *xym2*^b^	0.20 ± 0.04	0.02	This study	67%	100%	63%
*Piromyces sp.*^b,c^	0.35	0.07	This study	61%	63%	100%
*Piromyces sp.*	0.3 to 1.1	0.22	[18]	61%	63%	100%
*Piromyces sp*.^c^	0.5 to 0.8	0.02	[17]	61%	63%	100%
*Piromyces sp.*^c^	0.0538	0.056	[16]	61%	63%	100%
*Orpinomyces sp.*	1.6	0.01	[15]	61%	61%	94%
*Xanthomonas campestris*	0.016	0.02	[44]	64%	63%	61%
*Bacteroides thetaiotaomicron*	0.14	n.m.	[45]	61%	64%	83%
*Clostridium phytofermentans*^c^	0.0344	0.057	[16]	54%	52%	54%
*Clostridium thermosulfurogenes*	n.m.	n.d.	[46]	51%	49%	49%
*Escherichia coli*	n.m.	n.d.	[19]	50%	52%	48%
*Bacillus subtilis*	n.m.	n.m.	[47]	48%	48%	47%
*Lactobacillus pentosus*	n.d.	n.d.	[47]	48%	47%	46%
*Thermus thermophilus*	0.0007 to 0.012	n.m.	[48]	31%	30%	29%

^a^n.m., not measured; n.d., not detected. Sequence identity with *xym1*, *xym2 *and *Piromyces *XI gene is also given at the protein level. ^b^Glucose-grown cells. ^c^Codon-optimised gene.

*E. coli *has been the preferred choice as the host for screening metagenomic libraries [6] because numerous molecular tools are available and *E. coli *genetics are well elucidated. In our case, *E. coli *had the additional advantage that it is naturally able to utilize xylose, allowing the isolation of the desired activity without any other limitation up- or downstream of XI. The use of only one host, however, may limit the exploitation of microbial diversity for a given habitat due to, for instance, differences in codon utilization and/or mRNA and protein stability among different hosts. *E. coli *was, for instance, estimated to express only 40% of genes from diverse microbial origin [36]. In a previous study where growth-based screening was utilized for the isolation of novel D-3-hydroxybutyrate short-chain dehydrogenases from the soil metagenomic library, 25 positive clones were initially isolated when the library was screened in *Sinorhizobium melioti*; however, only one clone complemented the same phenotype in *E. coli *[32]. Screening of a soil metagenomic library performed in six different bacterial hosts identified only one case where the molecule-producing clone could be isolated using two different hosts [37].

Further screening of the soil metagenomic library described in this study should be performed in *S. cerevisiae *to investigate more suitable XIs for ethanol production from xylose. This is complicated by the fact that, whereas in bacterial metagenomic libraries large fragments are cloned and theoretically all genes within the fragment are transcribed and translated, only the closest gene to the methylated cap on the 5' terminus of the mRNA is translated in yeast [38]. Construction of future libraries for use in yeast will have to combine the cloning of small fragments corresponding to single gene size with an efficient transformation protocol to accommodate the larger colony numbers required to represent the same soil population. Alternatively, construction and screening of metagenomic cDNA libraries may be attempted; however, neither of these strategies has yet been reported.

## Conclusions

In the present study, a soil metagenomic library was successfully constructed and screened for the first time by using sequence- and growth-based methods to isolate genes conferring xylose isomerase activity. It enabled the isolation of *xym1 *and *xym2 *genes, respectively, that shared 67% identity at the protein level. Both genes could restore growth in *E. coli *and *S. cerevisiae *strains lacking the initial xylose catabolic pathway. However, the limited growth rates in *S. cerevisiae *highlight the importance of screening for XI activity in this host instead.

## Methods

### Plasmids and strains

Plasmids and strains used in this study are listed in Table 1. Strains were stored as recommended by the manufacturer or as 20% glycerol stocks in liquid media at -80°C.

### Cultivation conditions

*E. coli *strains were plated in Luria broth (LB) media plates (10 g/L Tryptone, 5 g/L yeast extract and 10 g/L NaCl, agar 15 g/L) and grown at 37°C overnight. Antibiotics were added when necessary to a final concentration of 30 μg/mL kanamycin and 50 μg/mL ampicillin and streptomycin. Single *E. coli *colonies were inoculated in LB media (10 g/L Bacto Tryptone, 5 g/L yeast extract and 10 g/L NaCl) for approximately 12 hours at 37°C in an incubator with constant agitation at 180 rpm (Boule Medical AB, Stockholm, Sweden).

For the metagenomic library screening, cells were plated in defined SM3 media [39] supplemented with 11 g/L xylose, 15 g/L agar, 1 mM isopropyl-β-D-thiogalactoside (IPTG) and 50 μg/ml ampicillin.

*S. cerevisiae *strains were grown on yeast nitrogen base (YNB) media plates (6.7 g/L Difco YNB without amino acids; Becton Dickinson, Sparks, MD, USA) supplemented with 20 g/L glucose and 20 g/L agar.

For growth kinetics on xylose, *E. coli *recombinant strains were pregrown in 20 mL of LB media at 180 rpm and 30°C until the end of the exponential phase. Cells were washed in distilled water and transferred to 500-mL Erlenmeyer flasks containing 50 mL of SM3 media supplemented with 30 g/L xylose and 1 mM IPTG at a starting OD_620nm _= 0.1.

*S. cerevisiae *strains were pregrown in 500-mL Erlenmeyer flasks containing 50 mL of YNB medium supplemented with 20 g/L glucose at 180 rpm and 30°C for approximately 12 hours. Cells were washed in distilled water and inoculated at OD_620nm _= 0.2 in 100 mL of YNB medium supplemented with 50 g/L xylose in 1-L Erlenmeyer flasks and grown at 180 rpm and 30°C.

### Standard molecular procedures

Standard DNA manipulation techniques were used [40]. Restriction and ligation enzymes were purchased from Fermentas (St. Leon-Rot, Germany). Primers were purchased from Eurofins MWG Operon (Ebersberg, Germany). DNA extraction from agarose gels and purification of PCR products were performed using the QIAquick extraction kit (Qiagen, Hilder, Germany). Plasmid DNA was prepared using the Bio-Rad Miniprep Kit (Bio-Rad, Hercules, CA, USA). Sequencing was performed at Eurofins MWG Operon. If not otherwise stated, bacterial transformation was performed by heat shock with competent cells prepared by using the Inoue Method for Preparation and Transformation of Competent *E. coli *as previously described [41]. Yeast transformation was performed as previously described [42].

### Construction of the soil metagenomic library

Soil DNA was extracted from the upper layer of a garden in Höör, southern Sweden, during Spring 2008. DNA extraction was performed using the PowerSoil DNA Isolation Kit (MoBio, Carlsbad, CA, USA). Isolated DNA was digested with *Bam*HI and *Mbo*I. Agarose gel electrophoresis was performed for purification of DNA fragments ranging between 2.0 and 6.0 kb to generate a small insert library with an average size of 4.0 kb. Vector pRSETB was digested with *Bam*HI and treated with alkaline phosphatase to prevent plasmid self-ligation. DNA fragments were ligated into pRSETB. Bacterial transformation was performed with ElectroTen-Blue competent cells (Stratagene, La Jolla, CA, USA) according to the manufacturer's instructions. The library size was estimated on the basis of the total number of *E. coli *clones minus the ones with empty plasmid.

### Cloning and expression of *xym1*, *xym2 *and XI from *Piromyces sp*

The isolated isomerase genes were cloned both into *E. coli *and *S. cerevisiae *expression vectors. The *xym1 *gene was amplified with primers XYM1F and XYM1R, which contain restriction sites for *Bam*HI and *Sal*I, respectively (Table 2). After PCR, both the amplified gene and plasmids pRSETB and p426TEF were digested. Plasmids with inserts obtained after ligation and bacterial transformation were sequenced and named pRSETB-Xym1 and p426TEF-Xym1, respectively (Table 1). Cloning of the *xym2 *gene followed the same procedure as for *xym1*, with the exception that *Eco*RI and *Sal*I restriction sites were used. *xym1 *and *xym2 *sequences will be publicly available when the corresponding patent application is published.

A codon-optimised version of the XI-encoding gene from *Piromyces sp. *was amplified by PCR utilizing plasmid YeplacHXT-XI as template [26]: forward primer 5' GGA*GGATCC*ATGGCTAAGGAATATTTTCCACAAATTC 3' and reverse primer 5' TTG*GAATTC*TTACTGATACATTGCAACAATAGCTTCG 3'. Restriction sites for *Bam*HI and *Eco*RI are underlined in the forward and reverse primers, respectively. Plasmid p426TEF [43], pRSETB (Invitrogen, Carlsbad, CA, USA) and the PCR product were digested with *Bam*HI and *Eco*RI and then ligated. Clones with inserts were sequenced prior to yeast transformation.

### Enzyme activities

*S. cerevisiae *strains were grown in YNB medium supplemented with 20 g/L glucose at 30°C to the early stationary phase. *E. coli *strains were pregrown in LB media supplemented with 100 μg/ml ampicillin for about 5 hours. After that, cells were washed with water and inoculated for an initial OD_620 nm _= 0.2 in SM3 medium supplemented with 10 g/L glucose and 1 mM IPTG at 37°C overnight.

Yeast and bacteria strains were harvested by centrifugation at 5,000 × *g *for 5 minutes and washed once with distilled water. Wet cells were suspended (0.5 mg/mL) in Y-per yeast or B-per bacteria extraction protein reagent (Pierce Biotechnology, Rockford, IL, USA) in a 2-mL Eppendorf tube and incubated on a turning table at room temperature for 50 minutes. Cell debris was spun down for 5 minutes at 16,100 × *g *(Z160M table centrifuge; HERMLE Labortechnik, Wehingen, Germany). Protein concentration was determined using Coomassie Protein Assay Reagent (Pierce Biotechnology). Bovine serum albumin was used to determine the standard curve.

XI activity was determined at 30°C using sorbitol dehydrogenase as previously described [18]. Assays were performed in a U-2000 spectrophotometer (Hitachi, Tokyo, Japan). Enzyme assays were performed in three biological replicates.

## Competing interests

The authors declare that they have no competing interests.

## Authors' contributions

NSP planned and carried out the experimental work and wrote the manuscript. MFGG participated in the study design and its coordination and critically read and commented on the manuscript. Both authors reviewed and approved the final version of the manuscript.
